# AI-powered precision in dental radiographic analysis using tailored CNNs for tooth numbering and cavity detection

**DOI:** 10.1371/journal.pdig.0001074

**Published:** 2025-11-20

**Authors:** Breno Guerra Zancan, José Andery Carneiro, Caio Uehara Martins, Camila Tirapelli, Camila Porto Capel, Eliana Dantas da Costa, Hugo Gaêta-Araujo, José Augusto Baranauskas, Alessandra Alaniz Macedo

**Affiliations:** 1 Department of Computing and Mathematics, FFCLRP, University of São Paulo (USP), Ribeirão-Preto, São Paulo, Brazil; 2 Department of Dental Materials and Prosthodontics, FORP-USP, Ribeirão-Preto, São Paulo, Brazil; 3 Division of Oral Radiology, Department of Stomatology, Public Oral Health and Forensic Dentistry, FORP-USP, Ribeirão-Preto, São Paulo, Brazil; National Yang Ming Chiao Tung University, TAIWAN

## Abstract

In the healthcare domain, images play a pivotal role in clinical diagnoses, treatment planning, surgical procedures, and epidemiological insights. Nevertheless, challenges such as limited experience among healthcare professionals, risk of misdiagnosis and subjective interpretation, and factors like stress and fatigue may jeopardize the precision with which patients are assessed. In this regard, professionals in the field of Dentistry face analogous challenges given that distinguishing anatomical structures in dental imaging requires expert interpretation and precise analysis. Convolutional Neural Networks (CNNs) offer promising opportunities to analyze images during patient care and can enhance diagnostic accuracy and clinical decision-making, benefiting both patients and healthcare providers. Here, we aimed to develop a specialized analyzer for **digital dental radiography**, that focuses on **numbering teeth and detecting tooth cavities**. The system is designed to achieve **high precision, recall, accuracy, specificity, and F1-score**, to ensure that diagnosis is reliable and accurate. In this study, we specifically explore Inception-v3 and InceptionResNet-v2 to discern cavitated teeth and tooth positions in dental panoramic radiographic images (PANs). On the basis of 935 PANs sourced from routine patient care, annotated by dentists at the Faculty of Dentistry of Ribeirão Preto in Brazil, our approach achieved precision of 0.98, recall of 0.98, accuracy of 0.998, specificity of 0.999 and F1-score of 0.98 for tooth numbering. Concerning identification of cavitated teeth, our approach reached precision of 0.96, recall of 0.91, accuracy of 0.94, specificity of 0.96 and F1-score of 0.94. By addressing the critical challenges and reaching high performance, our study serves as a benchmark that relates innovative research and real-world applications, fostering advancements in dental diagnosis. The performance reported herein demonstrates that our initiatives can modulate image analysis tasks and select a more suitable CNN for the job.

## Author summary

Dental radiographs are critical for diagnosing oral diseases, but interpreting them accurately can be challenging due to human factors like fatigue, experience, and complex anatomy. In our work, we developed a computer system that can automatically examine panoramic dental X-rays. Using nearly a thousand images carefully labeled by dentists, our system can recognize and number each tooth and detect cavities. We found that the system performs these tasks with high accuracy, showing that artificial intelligence can help reduce human error, save time in clinical practice, and provide more consistent assessments. By integrating intelligent image analysis into everyday care, our approach can support dental professionals in making faster, clearer, and more informed decisions.

## 1. Introduction

Dental and medical professionals are committed to delivering high-quality healthcare services, aiming to improve patient outcomes and overall quality of life. In Dentistry, radiographic images play an essential role in assessing the condition of teeth, gums, jaws, fractures, and bone structures [1,2]. These images are indispensable for detecting and diagnosing dental issues early, before they progress to advanced stages and severe consequences. Although dental panoramic radiographs are limited in terms of resolution, contrast, and structural superimposition, they remain valuable for identifying the extent of cavitated carious lesions and numbering teeth, among other tasks. Moreover, radiographic images are essential for treatment planning and surgical procedures because they allow hidden dental structures, benign or malignant masses, bone loss, periodontal problems, impacted teeth, and cavities to be identified [3,4].

Despite the invaluable support provided by radiographs, several factors, including a dental care professionals’s limited experience, subjective interpretation, stress, and fatigue, pose challenges when it comes to accurately assessing the patients’ oral conditions [5–8]. Computational technologies accelerate tasks like classifying images, detecting diseases, and numbering teeth, reducing human error due to workload overload. Convolutional Neural Networks (CNNs) and Deep Learning (DL) have been successful, in healthcare areas where images are a crucial information source to identify and to classify pulmonary nodules automatically [9,10], interpreting mammograms when screening cancer [11], detecting liver diseases [12], and identifying melanomas and malignant carcinomas [13], among other tasks.

In Dentistry, promising progress has been made in automated caries detection in intraoral radiographs like scanner [14], bitewing [15–20], and periapical [21–29] dental images. Lee et al. focused on detecting decayed teeth in periapicals and achieved average accuracy of 0.84 [23]. Bouchahma et al. manipulated panoramic and periapical dental images to detect fluoride, filling and root canal treatments with overall accuracy of 0.87 [26]. Zhang et al. manipulated intraoral images to number teeth [24]. However, extraoral radiographs offer an opportunity to explore a comprehensive view of all teeth in a single image, facilitating not only tooth numbering [30–36], but also other tasks in digital dentistry [37–43].

Usually, non-cavitated or initial caries lesions are not easily discernible to the human eye on tooth radiographs. This limitation stems from the narrow range of grayscale shades that the human eye can distinguish. In contrast, a high-performance computational system can perceive distinct shades of gray as a numerical value and learn to detect patterns, thus expanding diagnostic possibilities. Verma et al. successfully identified various dental issues, such as cavities, infections, and impacted teeth, when they used advanced computational techniques to explore panoramic radiographs [44]. Similarly, Lian et al. applied these methods to panoramic radiographs and detected and classified dental cavities [45].

Briefly, our focused problem statement highlights the challenges in interpreting dental panoramic radiographs (PANs) due to subjective assessment, variable clinical experience, and human visual limitations. While DL has been employed in medical imaging, its application in automated tooth numbering and tooth cavitation - which in some cases can indicate installed dental caries - in PANs remains underexplored. To address this, we present a two-phase method to analyze PANs by using DL to identify cavitated teeth and to number tooth positions, according to the FDI World Dental Federation notation. We have delved into using two CNN architectures, namely Inception-v3 and Inception-ResNet-v2, to tackle tooth numbering and to identify cavitated teeth within PANs. The architectures have been designed to identify all 32 teeth on the basis of the FDI notation. Regarding cavitated tooth identification, we have combined data from two datasets to train the CNNs with a view to enhancing their performance. The core contribution of our study lies in a modular application with specialized modules, with each module contributing to the overall effectiveness of the method. We intend to alleviate the dental healthcare professionals’ workload and to provide dentists with valuable and accurate support during diagnosis.

## 2. Materials and methods

In the hope that our research will benefit users, here we have conducted a proof of concept that manipulates dental images, assigns numbers to teeth, and identifies cavitated teeth in dental PANs, aiming to achieve high precision, recall, accuracy, specificity, and F1-score [46]. To accomplish this, we evaluated different CNN architectures on the basis of performance criteria to determine how effectively they analyze dental images. Our ultimate objective was to create specialized modules to segment and to number teeth, and to detect tooth cavities, so as to set a benchmark for relating innovative research and real-world applications to processing dental radiographs reliably. Thus, we created components by considering the following: (Task 1) *detecting the mouth region*; (Task 2) *segmenting each tooth*; (Task 3) *assigning FDI numbers to the segmented teeth*; and (Task 4) *identifying cavitation in the crown of teeth*. Tasks 1 and 2 were developed in [47], as part of a modular architecture, and their output served as input for Tasks 3 and 4, presented in this study. Briefly, Task 1 detected the dentomaxillo region (encircling mandible, maxilla and teeth in a bounding box), and Task 2 outlined the instance segmentation of the teeth by using only the dentomaxillo region. For Tasks 1 and 2, we manipulated 935 PANs stored in their original JPEG format. During these phases, we kept the PAN dimensions at 2903 x 1536 pixels and resolution at 300 dpi, but we did not make adjustments, such as brightness, contrast, cropping, or resizing.

Task 3 involved assigning FDI numbers to the teeth segmented in Task 2. To train and to test this task, we used 18,836 dental images sourced from the 935 PANs. For Task 4, which focused on identifying cavitated-teeth, we used the FDI-numbered teeth from Task 3 as input. To train and to test this task, we worked with 7,304 dental images that depicted both cavitated and non-cavitated teeth and which had been obtained from 1,113 PANs across two datasets.

Dentists rely on tooth numbering systems to pinpoint and to discuss specific teeth. Knowing the number of a cavitated tooth is critical for various reasons: (a) it aids treatment planning and enables personalized approaches according tooth location and type; (b) it helps to monitor the prevalence of active dental caries and to design preventive measures and timely interventions; (c) it facilitates accurate referrals to specialists, ensuring that patients receive proper care; (d) and it maintains comprehensive dental records, allowing for documentation to be organized and being useful for future treatment planning and referrals. Overall, knowing the number of the cavitated tooth is key to effective dental diagnosis and treatment and ongoing oral health management.

**Fig 1:**
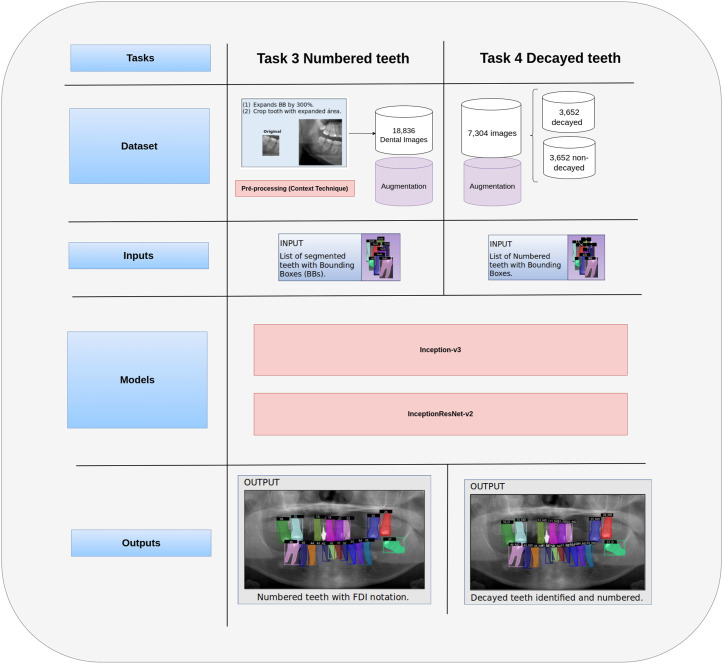
Illustration of Task 3 (left) and Task 4 (right), which refer to the numbering modules for identifying decayed teeth. The input to the numbering module (Task 3) comprises the segmented teeth (from Task 2). After that, the teeth are numbered and submitted to cavitated tooth identification (Task 4).

Fig 1 illustrates the modules for Task 3 and Task 4, which focus on numbering teeth and identifying cavitated teeth, respectively. Task 3, “Numbered Teeth,” refers to the FDI numbering system. In this task, each tooth image is expanded to include its surrounding context, which generates a new image. After that, data augmentation is applied to these new images to diversify the dataset. Therefore, the augmented dataset is used as input for trained architectures, either Inception-v3 or InceptionResNet-v2, to assign tooth numbers, so that the performance of the two architectures can be compared. Finally, the output consists of teeth numbered according to the FDI system. For Task 4, the number of images of cavitated and non-cavitated teeth is balanced to create the test dataset, which is fed into two trained architectures, Inception-v3 and InceptionResNet-v2, to determine whether a tooth has cavities. This allows the performance of the architectures to be compared. As a result, the output is a set of numbered teeth and indicates whether cavities are present or absent.

The next subsections describe the datasets we used to number teeth and to identify cavitated teeth, the metrics we applied to evaluate architectures, and the outcomes of these assessments, and the eligibility criteria that sustain the selection of architectures. The next section details the tasks of tooth numbering and cavitated tooth identification. Our research underwent thorough ethical review, received approval from the local Research Ethics Committee (Brazil Platform CAAE: 51238021.2.0000.5419), and adhered to the principles of the Declaration of Helsinki.

### 2.1. Data acquisition

Our approaches explored our dataset, FD; and a public dataset, PD. The FD dataset comprises 935 PANs obtained as part of the routine care of 935 patients aged at least 18 who were seen at the Faculty of Dentistry of Ribeirão Preto (FORP), University of São Paulo, Brazil, from January 2014 to January 2020. The patients signed an informed consent form. From July to September 2020, a qualified radiologist meticulously selected high-quality PANs of permanent teeth, ensuring no repetitions, from FORP’s extensive clinical image database to compose the FD dataset. Images with baby teeth, abnormalities, damage, or artifacts were excluded to ensure a clean dataset for research. Next three dentists labeled 935 PANs, identified 18,836 teeth and labeled them according to the FDI system, and they classified 885 teeth as decayed. The dataset reflects a natural class imbalance inherent to human dental anatomy and differences in oral health status found across different groups within the treated population. Certain teeth, such as third molars, are less common due to factors like agenesis or extraction, while others, such as canines, tend to appear less frequently in panoramic radiographs due to anatomical or positioning variations. This distribution accurately reflects real population conditions and poses challenges for classification tasks.

To ensure annotation quality and consistency, we implemented an annotation protocol and a multi-expert review process. The protocol was developed and refined through regular meetings with a multidisciplinary team—including radiologist, dentists, and computing experts—ensuring consistency, clarity, and reproducibility across the dataset. All 935 panoramic radiographs were initially annotated by Radiologist 1, followed by independent verification by Radiologist 2. Discrepancies between the two annotators occurred in only 163 instances (0.7% of the total annotations) [48]. These cases were adjudicated by a third radiologist, ensuring consensus-based labeling. These results indicate a high degree of inter-rater reliability. Although formal inter-rater reliability metrics such as Cohen’s Kappa were not computed, the low disagreement rate and the consensus-based adjudication approach suggest strong agreement among experts. Tooth caries labeling was based on a protocol developed through collaboration between dental clinicians and radiologists. Annotators visually inspected each cropped tooth image for radiolucent areas on the enamel or dentin—characteristics consistent with carious lesions in panoramic radiographs. These criteria were derived from standard dental diagnostic conventions and adapted to the image quality and anatomical presentation of PANs. This protocol helped standardize caries identification across datasets and ensured consistency throughout the annotation process.

The FD dataset was published in [48] with DOI https://doi.org/10.1016/j.oooo.2023.12.006. All the image files are available at https://inredd.com.br/pt-BR/solutions/open-datathrough the proof of concept described later in this paper.

The PD dataset consists of PANs obtained in July 2023 with varying image quality annotated by dental experts. The images represent patients aged at least 12 years and were randomly selected from hospital databases. Privacy and confidentiality were ensured. Final-year dentistry students initially labeled each image, which was followed by rigorous review and correction by one of the three highly experienced (over 15 years of expertise) dentists mentioned above. The annotations detail the FDI number of each tooth and identify four key dental abnormalities: caries, deep caries, periapical lesions, and impacted teeth. The data is provided under the CC BY-SA 4.0 License, which ensures that it is completely open-source. For our experiments, we used teeth marked as having cavities at any stage. The PD dataset is available at https://github.com/ibrahimethemhamamci/DENTEX.

To investigate cavitated teeth, we added 2,767 images from the publicly available PD dataset to our original FD dataset, which contained 885 images of decayed teeth. This resulted in a total of 3,652 cavitated tooth images, enabling a more robust analysis of cavitated versus healthy teeth. Furthermore, we incorporated an equal number of non-cavitated teeth from the FD dataset to balance the two categories, cavitated and non-cavitated teeth, which amounted to 3,652 teeth in each category and a total of 7,304 images. The combination of PANs obtained from the FD dataset and the PD dataset from different countries helped to reduce bias in our experimental datasets, ensuring better demographic representation. In recent dental practice, particularly in triage settings, public health clinics, or low-resource regions, panoramic radiographs often represent the only imaging modality available for initial oral assessment [49,50]. Despite the superior diagnostic resolution of intraoral images for early caries detection, panoramic radiography remains prevalent due to its wide coverage of the entire dentition, its ability to be obtained more comfortably and rapidly, and cost-effectiveness for large-scale screening [50,51]. Panoramics also support automated workflows such as tooth segmentation, numbering, and multi-pathology detection, demonstrating considerable promise when integrated with AI models—even if sensitivity for caries detection remains lower than intraoral methods [52,53]. Therefore, while not intended to replace intraoral radiography, this study explores panoramic images as a pragmatic, accessible first step toward automatic tooth cavitations identification and dental chart generation.

Subsequently, we trained each architecture separately, according to their objective. For the tooth numbering task, we trained and tested two architectures, Inception-v3 and InceptionResNet-v2, by using 18,836 teeth annotated according to the FDI numbering. For the task of identifying cavitated teeth, we used 7,304 teeth, 3,652 of which were cavitated and 3,652 of which were non-cavitated, to train and to test Inception-v3 and InceptionResNet-v2. Regarding the class imbalance in both tasks, we avoided using synthetic oversampling techniques such as SMOTE or ADASYN, which may generate unrealistic interpolations in medical imaging tasks and compromise anatomical consistency [54,55]. Instead, task-specific augmentation strategies were adopted.

### 2.2. CNN architectures and evaluation

To select the most effective architectures for our tasks, we analyzed relevant research [17,23,35,40] from our systematic mapping [56] (This systematic literature review on “Deep Learning for Detecting and Classifying Teeth, Dental Caries, and Restorations” provided us with new insights into dataset characteristics, methodologies, and evaluation criteria and identified key patterns, gaps, and opportunities for future research. The reproducible results allow potential research directions to be further explored.) and explored publicly available information on different architectures provided by Keras API (refer to keras.io or Keras in TensorFlow www.tensorflow.org for creating and training DL models), which describes how the model performs on the ImageNet validation dataset. Studies [35,40] have used ResNet-50 and ResNet-152 architectures to classify teeth/tooth numbering, with resolutions of 224x224 and 227x227, respectively. In contrast, here we chose the Inception-V3 and InceptionResNet-v2 architectures, which offer higher accuracy with fewer parameters than ResNet, as informed by Keras. Fewer parameters improve computational efficiency, generalization, interpretability, training time, and energy consumption. Two studies [17,23] have applied Inception-v1 and Inception-v3 to identify decayed teeth, with input resolutions of 130x20 and 299x299, respectively. Both these studies used intraoral radiographs, which clearly show decayed teeth. Using intramural radiographs made their task easier than using PANs. Following the approach of studies [15,21], we also used the Inception-v3 and InceptionResNet-v2 architectures herein, with references from the ImageNet dataset. We considered established methods while also being open to exploring promising new developments in the field. Moreover, we employed a custom DL framework constructed with refactored Python code by using the Keras API. This framework enabled two architectures to be created and trained for both tooth numbering and cavitated tooth identification tasks. Besides that, it leveraged transfer learning with pre-trained weights from the ImageNet dataset (https://www.image-net.org/about.php).

To tailor the architecture, we defined hyperparameters such as the number of epochs, batch size, and dropout and learning rates. Additionally, we applied data augmentation techniques, including PAN vertical and horizontal flips, rotation, brightness adjustments, and zoom variations. We resized all the images to 299 × 299 pixel resolution as input in the CNNs to achieve computational efficiency. This reduced the training time and enabled downsampling for standardization given that a well-trained and fine-tuned CNN can still extract salient features effectively even though higher resolutions retain more details.

Our work involves different types of measurements. Some are specific to detection (Task 1), while others such as IoU, pixel accuracy, and Hausdorff Distance are specific to segmentation (Task 2). To number teeth (Task 3) and to identify cavitated teeth (Task 4), we specifically used accuracy, recall, precision, and F-measure, which are the most frequently employed metrics according to our systematic mapping presented in [56].

We applied a common technique called five-fold cross-validation to evaluate our machine learning models. We split our dataset (E) into five roughly equal groups (F1-F5) and ensured that no image appeared twice, and that class distribution (e.g., tooth types) was maintained. Then, we ran the experiment five times. Each time, one group (FT) was set aside for testing and validation (further split in half), while the remaining four groups were used for training. To rigorously compare the performance of the Inception-v3 and InceptionResNet-v2 models across both classification tasks—namely, tooth identification by FDI numbering (32 classes) and binary detection of cavitated vs. non-cavitated teeth—we employed the Wilcoxon signed-rank test. This non-parametric test was chosen because it is suitable for paired data (e.g., the same tooth class evaluated across folds for each model) and does not assume normality, which is appropriate given the small number of folds (n = 5) [57]. Since each task involved multiple statistical comparisons (across classes and metrics), we applied the Benjamini-Hochberg False Discovery Rate (FDR) correction to control the expected proportion of false positives and reduce Type I error inflation [58]. This combination provides a statistically robust and conservative framework for determining whether observed differences between models are meaningful or due to random variation.

We conducted the experiments on the Python on Google Collaboratory (Colab) platform, which offers Ubuntu OS, 12.7 GB RAM, 168.8 GB disk space, and a free 15 GB NVIDIA Tesla T4 GPU and CUDA 12.

## 3. Experiments, results and discussion

To configure the hyperparameters for both tasks—assigning FDI numbers to teeth and identifying cavitated teeth—we based the initial settings on default values from the Keras library, specifically a learning rate of 0.001 and a batch size of 32. We selected the Adam optimizer, a first-order gradient-based optimization algorithm designed for stochastic objective functions, because it has adaptive learning rate properties. Adam is widely used in deep learning applications since it offers computational efficiency with minimal memory requirements and has proven effective across various tasks, including classifying images, detecting objects, and segmenting teeth by using CNNs [59,60]. However, its performance can vary depending on the specific task and dataset. While we executed the model, we monitored key metrics such as loss function, precision, recall, and accuracy, for both training and validation. We applied an empirical early stopping strategy: if the validation loss did not improve for three consecutive epochs, we halted training. Additionally, if the validation loss did not increase after a single epoch, we reduced the learning rate by half. After we established these criteria, we fine-tuned batch size values through empirical adjustments, referencing values from foundational research. This iterative process ultimately led to the final set of hyperparameters we used in this study.

### 3.1. Tooth numbering

**Fig 2:**
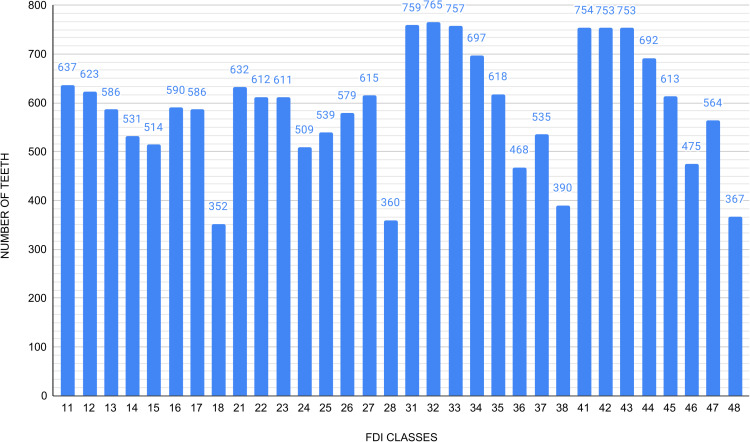
Number of teeth by each FDI notation.

We trained and evaluated Inception-v3 and InceptionResNet-v2 by using Keras to number teeth (Task 3). The dataset consisted of 18,836 dental images, from FD, which we employed for both training and testing. The quantities of these teeth according to the FDI numbering system are presented in Fig 2 The dental group with the largest number of images was the molar group (5,881), which corresponds to the sum of teeth 16, 17, 18, 26, 27, 28, 36, 37, 38, 46, 47 and 48; followed by the incisor group (5,535), which corresponds to the sum of teeth 11, 12, 21, 22, 31, 32, 41 and 42; the premolar group (4,713), which corresponds to the sum of teeth 14, 15, 24, 25, 34, 35, 44 and 45; and the canine group (2,707), which corresponds to the sum of teeth 13, 23, 33 and 43. The dataset used presents a natural class imbalance, as certain teeth, such as third molars, occur less frequently in the population and are therefore underrepresented in panoramic radiographs. This asymmetry may impact model performance by favoring more common classes.

To improve the performance of our architectures and mitigate overfitting, while also addressing the natural class imbalance in the dataset, we adopted a multi-step strategy for the experiments. The bounding boxes previously annotated by specialists were expanded by 300% in both height and width. This enlargement was intended to include the anatomical neighborhood of each tooth, allowing the neural network to process not only the target tooth but also its surrounding context, as inspired by [61]. Based on these context-enriched images, data augmentation techniques were applied using the ImageDataGenerator class from the Keras library. The ImageDataGenerator applies random transformations in real time to each batch, ensuring that, in every epoch, the model is exposed to novel variations of the original images — a widely accepted strategy to reduce overfitting through augmentation-based regularization. The selected augmentation parameters were carefully chosen to reflect realistic radiographic acquisition scenarios: a 25% horizontal shift simulated lateral displacements of the dental arch caused by minor head tilts or patient movement; a 25% vertical shift represented variations in head positioning relative to the horizontal X-ray beam, altering the perceived height of the arch; a 15% zoom captured changes in the distance between the patient and the sensor, or digital magnification differences; and the “nearest” fill mode preserved anatomical continuity by avoiding artificial pixel artifacts in transformed areas. These transformations increased dataset diversity and realism, improving generalization and reducing the risk of overfitting.

Although data augmentation alone does not fully resolve class imbalance, it contributed to model robustness by simulating plausible positional and scale variations. Furthermore, model performance was evaluated using imbalance-sensitive metrics such as the macro F1-score and confusion matrix, which are less influenced by majority classes. While cost-sensitive learning strategies (e.g., weighted loss functions) were considered, they were not implemented in this study. Instead, the focus was on anatomical context enhancement and augmentation-driven generalization. In particular, canine teeth were noticeably underrepresented, further highlighting the anatomical origin of the imbalance and the importance of mitigation strategies tailored to class prevalence and radiographic visibility.

We investigated different hyperparameters to configure Inception-v3 and InceptionResNet-v2 for tooth numbering in panoramic radiographs based on the FDI system. The number of training epochs was set between 30 and 45 for Inception-v3 and between 15 and 25 for InceptionResNet-v2, based on empirical observations of convergence behavior and signs of overfitting in preliminary tests. InceptionResNet-v2 required fewer epochs due to its residual connections, which accelerate convergence. A batch size of 16 was selected for both models as a trade-off between training stability and memory constraints of the available GPU. Dropout and learning rate values were set individually for each model after testing several combinations and monitoring validation performance. For Inception-v3, we used a higher dropout rate (0.6) and a lower learning rate (0.0001) to counterbalance its tendency to overfit when trained on smaller datasets, as suggested in prior literature and open-source repositories. For InceptionResNet-v2, we used a lower dropout (0.2) and a higher learning rate (0.001), allowing the model to learn more aggressively while still benefiting from residual regularization. These choices were informed by a combination of values recommended in publicly available code bases (e.g., GitHub projects for medical imaging classification using Inception-based architectures), findings in related studies, and a series of ad-hoc experiments in which different settings were tested and evaluated based on validation accuracy, F1-score, and signs of early overfitting.

Table 1 lists the results of precision, recall, accuracy, specificity and F1-score for each dental class that represents a specific FDI number, which resulted from the tests for numbering teeth performed with Inception-v3 and InceptionResNet-v2. This table shows the arithmetic mean of the tests performed with the architectures obtained with five-fold cross-validation; the last row shows the averages of the column values.

**Table 1:** All results of Inception-v3 and InceptionResNet-v2, results regarding tooth numbering.

C	Inception-v3					InceptionResNet-v2				
	Pre	Rec	Acc	Spe	F1	Pre	Rec	Acc	Spe	F1
11	0.973	0.985	0.999	0.999	0.978	0.997	1.000	1.000	1.000	0.998
12	0.965	0.949	0.997	0.999	0.957	0.997	0.987	0.999	1.000	0.992
13	0.935	0.963	0.997	0.998	0.948	0.980	0.990	0.999	0.999	0.985
14	0.915	0.883	0.994	0.998	0.898	0.981	0.962	0.998	0.999	0.971
15	0.912	0.883	0.994	0.997	0.898	0.962	0.988	0.999	0.999	0.975
16	0.963	0.919	0.996	0.999	0.939	0.990	0.969	0.999	1.000	0.979
17	0.867	0.946	0.994	0.995	0.903	0.957	0.963	0.997	0.999	0.959
18	0.925	0.874	0.996	0.998	0.892	0.951	0.960	0.998	0.999	0.955
21	0.991	0.978	0.999	1.000	0.984	0.997	1.000	1.000	1.000	0.998
22	0.978	0.967	0.998	0.999	0.972	1.000	1.000	1.000	1.000	1.000
23	0.959	0.974	0.998	0.999	0.966	0.987	0.993	0.999	1.000	0.990
24	0.939	0.887	0.995	0.998	0.909	0.984	0.973	0.999	1.000	0.978
25	0.899	0.912	0.995	0.997	0.905	0.993	0.989	0.999	1.000	0.991
26	0.941	0.904	0.995	0.998	0.920	0.983	0.983	0.999	0.999	0.983
27	0.873	0.919	0.993	0.995	0.861	0.974	0.952	0.998	0.999	0.962
28	0.908	0.906	0.996	0.998	0.905	0.937	0.972	0.998	0.999	0.954
31	0.891	0.835	0.989	0.996	0.861	0.992	0.984	0.999	0.999	0.984
32	0.855	0.901	0.990	0.993	0.877	0.992	0.982	0.999	1.000	0.987
33	0.912	0.948	0.994	0.996	0.928	0.992	1.000	1.000	1.000	0.996
34	0.929	0.946	0.995	0.997	0.937	0.992	0.994	0.999	1.000	0.993
35	0.953	0.907	0.995	0.998	0.929	0.994	0.981	0.999	1.000	0.987
36	0.921	0.920	0.996	0.998	0.920	0.983	0.979	0.999	1.000	0.981
37	0.877	0.900	0.993	0.996	0.885	0.953	0.963	0.998	0.999	0.958
38	0.901	0.903	0.996	0.998	0.895	0.961	0.959	0.998	0.999	0.959
41	0.950	0.830	0.991	0.998	0.883	0.979	0.987	0.999	0.999	0.983
42	0.899	0.942	0.993	0.995	0.919	0.989	0.976	0.999	1.000	0.983
43	0.948	0.958	0.996	0.998	0.953	0.987	0.997	0.999	0.999	0.992
44	0.943	0.922	0.995	0.998	0.931	0.997	0.983	0.999	1.000	0.990
45	0.908	0.935	0.995	0.997	0.920	0.984	1.000	0.999	0.999	0.992
46	0.923	0.850	0.994	0.998	0.881	0.983	0.979	0.999	1.000	0.981
47	0.870	0.922	0.993	0.995	0.891	0.944	0.954	0.997	0.998	0.949
48	0.924	0.903	0.997	0.998	0.911	0.946	0.925	0.997	0.999	0.935
–	0.923	0.918	0.995	0.997	0.917	0.979	0.979	0.999	0.999	0.979

We trained the architectures by using 32 dental classes, referring to the positions of each tooth, identified with the FDI numbering. Table 1 shows that the InceptionResNet-v2 achieved better results in all metrics, in all classes. Molars 17, 18, 27, 28, 37, 38, 45, and 48 had the lowest F1-score for both architectures. One plausible explanation for this pertains to sample size. Third molars 18, 28, 38, and 48 had the smallest number of samples. For other molars, the influence of neighboring teeth could also be a contributing factor, given that they have the fewest neighbors in our dataset, trailing only behind third molars in this aspect [62]. The overall means of the results obtained with Inception-v3 for precision, recall, accuracy, specificity and F1-score were 0.923, 0.918, 0.995, 0.997 and 0.919, respectively. For these same metrics, we obtained 0.979, 0.978, 0.998, 0.999 and 0.978 with InceptionResNet-v2, respectively.

**Fig 3:**
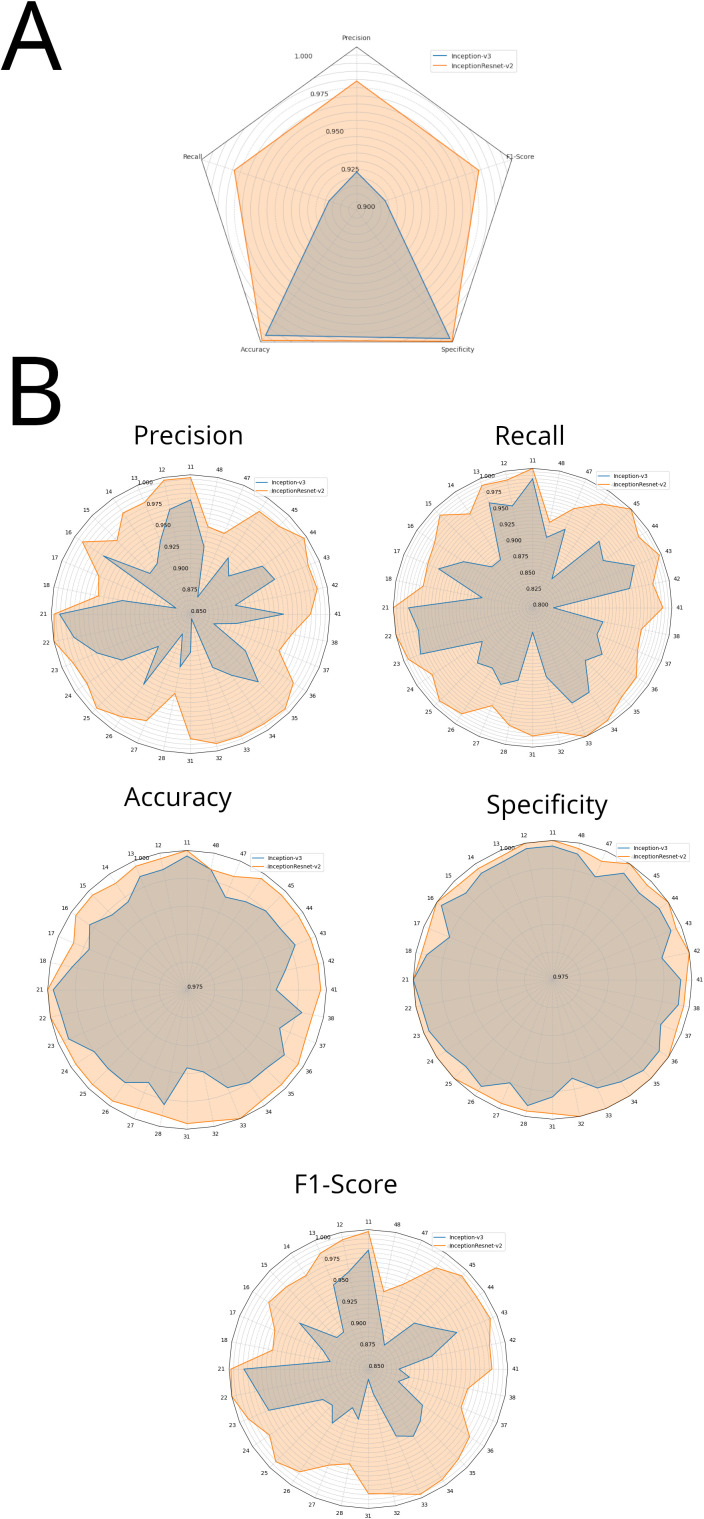
Performance of Inception-v3 and InceptionResNet-v2 in distinguishing 32 FDI classes (refer to Table 1). **(A)** Comparison of Inception-v3 and InceptionResNet-v2 average metrics **(B)** Each class of Inception-v3 and InceptionResNet-v2 metrics are compared.

Fig 3 illustrates the results of the tooth numbering tests conducted by using the Inception-v3 and InceptionResNet-v2 architectures. Fig 3A compares the average metrics of both architectures, while Fig 3B presents a detailed comparison of each metric across all 32 dental classes for both architectures. The detailed results are presented in Table 1.

According to the values obtained with five-fold cross-validation, we calculated the means, standard deviations (SD), and estimated minimum and maximum values for a 95% confidence interval (CI) for the metrics precision, recall, accuracy, specificity, and F1-score. Table 2 shows these values for the Inception-v3 and InceptionResNet-v2 architectures.

**Table 2:** Means, standard deviations and estimated values for a 95% confidence interval for Task 3 (tooth numbering) obtained for each architecture.

Metrics	Inception-v3				InceptionResnet-v2			
	Mean (x)	SD (σ)	95% CI		Mean (x)	SD (σ)	95% CI	
			Lower Limit	Upper Limit			Lower Limit	Upper Limit
Precision	0.9240	± 0.0061	0.9161	0.9311	0.9794	± 0.0045	0.9738	0.9850
Recall	0.9184	± 0.0084	0.9184	0.9288	0.9790	± 0.0037	0.9744	0.9836
Accuracy	0.9950	± 0.0007	0.9941	0.9959	0.9980	± 0.0004	0.9982	0.9994
Specificity	0.9976	± 0.0005	0.9969	0.9983	0.9992	± 0.0004	0.9986	0.9998
F1-score	0.9188	± 0.0081	0.9088	0.9288	0.9788	± 0.0043	0.9734	0.9842

InceptionResNet-v2 achieved higher average values than Inception-v3 across all metrics, suggesting a consistent trend toward improved performance in the classification of teeth based on FDI numbering. Specifically, InceptionResNet-v2 reached average values of 0.9794 for precision, 0.9790 for recall, 0.9988 for accuracy, 0.9992 for specificity, and 0.9788 for F1-score. These high values, combined with small standard deviations (e.g., SD = 0.0045 for precision, 0.0043 for F1-score), indicate stable results across the five folds of cross-validation. The corresponding 95% confidence intervals were narrow—for instance, ranging from 0.9738 to 0.9850 for precision and from 0.9744 to 0.9836 for recall—suggesting high reliability in these estimates. Furthermore, the nearly identical precision and recall values indicate balanced sensitivity and specificity, reflecting low false positive and false negative rates. Although InceptionResNet-v2 consistently achieved higher average values across nearly all metrics and classes when compared to Inception-v3, the Wilcoxon signed-rank tests revealed that none of the differences reached statistical significance after FDR correction (all p_FDR > 0.05). Some of the most prominent average improvements were observed in F1-score for mandibular molars (e.g., classes 36, 37, 38, 46, 47, 48), and in recall and precision for lower incisors and canines (e.g., classes 31, 32, 33, 41). Specificity remained high for both models but was slightly higher overall for InceptionResNet-v2. The absence of statistically significant differences is likely due to the limited number of folds (n = 5), which constrains statistical power, even though performance trends consistently favored InceptionResNet-v2.

While no individual comparison reached statistical significance after FDR correction, the consistent pattern of improvement with InceptionResNet-v2 suggests that this architecture offers a tangible advantage, particularly for anatomically challenging regions such as posterior teeth. The improved performance is likely due to the enhanced feature propagation and residual connections inherent in the InceptionResNet-v2 design. These findings highlight the importance of using multiple folds, class-wise metrics, and robust statistical validation when comparing deep learning architectures in medical imaging.

### 3.2. Identification of cavitated teeth

We investigated Inception-v3 and InceptionResNet-v2 by using Keras and a modified code from GitHub to identify cavitated teeth. We compared the same metrics on the two architectures. Both datasets contained PANs with teeth cropped on the basis of radiologist annotations. These cropped teeth served as input for the architectures.

We added 2,767 cavitated teeth images from the PD dataset to 885 cavitated teeth images from the FD dataset (3,652 samples). For non-cavitated teeth, we used 3,652 images from the FD dataset; heavily restored, broken, or pinned teeth were excluded. Hence, we had a balanced dataset of 7,304 images (3,652 decayed and 3,652 non-cavitated tooth images). Again, we used five-fold cross-validation for training and testing. We also applied data augmentation techniques (vertical/horizontal flip, rotation, brightness variation, and zoom variation) to enrich the training data. We empirically determined the values for each augmentation strategy.

Although class weighting was considered to mitigate this imbalance, it was not implemented in this experiment due to the binary nature of the classification task and the mitigating effect of extensive data augmentation.

To implement Inception-v3, we employed 37 training epochs, a batch size of 8, a dropout rate of 0.6, and a learning rate of 0.001. For InceptionResNet-v2, we used 35 training epochs, a batch size of 8, a dropout rate of 0.2, and the same learning rate of 0.001. These hyperparameters were selected empirically through iterative experimentation, with adjustments made based on validation loss, accuracy, and macro F1-score curves. The higher dropout rate for Inception-v3 (0.6) was used to mitigate overfitting, especially considering the relatively small dataset and the model’s tendency to memorize training data in complex classification tasks. In contrast, InceptionResNet-v2, which benefits from residual connections that help prevent overfitting, was trained with a lower dropout rate (0.2). A smaller batch size of 8 was chosen due to GPU memory limitations and to allow for more granular weight updates, which can help generalization in medical imaging tasks. Both models used a learning rate of 0.001, which showed stable convergence behavior during early training iterations. The learning rate was not decreased further to maintain a balance between training time and accuracy. All input images were resized to 299 × 299 pixels, which corresponds to the expected input dimensions for both CNN architectures and ensures compatibility with their pretrained weights when using transfer learning.

Table 3 displays the average values of the outcomes across the five-folds, categorized by individual classes as well as both classes combined. On average, recall was the lowest metric for cavitated and non-cavitated teeth. InceptionResNet-v2 had smaller differences in performance between the two classes than Inception-v3, so the former can distinguish between cavitated and non-cavitated teeth more accurately. Non-cavitated teeth were better recognized by both architectures. Finally, Inception-v3 gave 0.951, 0.866, 0.910, 0.960, and 0.910 for precision, recall, accuracy, specificity and F1-score, respectively. In turn, InceptionResNet-v2 provided 0.963, 0.914, 0.939, 0.965 and 0.937, for precision, recall, accuracy, specificity, and F1, respectively.

**Table 3:** Average values of the outcomes across the five folds, to identify cavitated teeth.

	Inception-v3					InceptionResNet-v2				
C	Pre	Rec	Acc	Spe	F1	Pre	Rec	Acc	Spe	F1
N-dec	0.991	0.915	0.950	0.990	0.951	0.991	0.937	0.962	0.991	0.963
Dec	0.910	0.818	0.870	0.930	0.860	0.934	0.891	0.915	0.938	0.911
N-Dec/Dec	0,951	0.866	0.910	0.960	0.910	0.963	0.914	0.939	0.965	0.937

On the basis of the values obtained with five-fold cross-validation for the task of identifying cavitated teeth, we calculated the means, standard deviations (SD) and estimated minimum and maximum values for a 95% confidence interval (CI) for the metrics precision, recall, accuracy, specificity, and F1-score. Table 4 shows the values for the Inception-v3 architecture, whereas Table 5 shows the values for the InceptionResNet-v2 architecture.

**Table 4:** Means, standard deviations and estimated values for a 95% confidence interval, resulting from the Inception-v3 architecture for Task 4 (decayed tooth identification).

Inception-v3								
Metrics	Non-decayed class				Decayed class			
	Mean (x)	SD (σ)	95% CI		Mean (x)	SD (σ)	95% CI	
			Lower Limit	Upper Limit			Lower Limit	Upper Limit
Precision	0.9910	± 0.0008	0.9902	0.9922	0.9102	± 0.0566	0.8400	0.9804
Recall	0.9154	± 0.0618	0.8386	0.9922	0.8166	± 0.0242	0.7866	0.8466
Accuracy	0.9494	± 0.0331	0.9083	0.9905	0.8710	± 0.0140	0.8536	0.8884
Specificity	0.9900	–	–	–	0.9296	± 0.0565	0.8594	0.9998
F1-score	0.9508	± 0.0346	0.9078	0.9938	0.8602	± 0.0159	0.8404	0.8800

**Table 5:** Means, standard deviations and estimated values for a 95% confidence interval, resulting from the InceptionResNet-v2 architecture for Task 4 (decayed tooth identification).

InceptionResNet-v2								
Metrics	Non-decayed class				Decayed class			
	Mean (x)	SD (σ)	95% CI		Mean (x)	SD (σ)	95% CI	
			Lower Limit	Upper Limit			Lower Limit	Upper Limit
Precision	0.9914	± 0.0005	0.9907	0.9921	0.9334	± 0.0255	0.9017	0.9651
Recall	0.9370	± 0.0270	0.9034	0.9706	0.8908	± 0.0263	0.8582	0.9234
Accuracy	0.9622	± 0.0139	0.9449	0.9795	0.9148	± 0.0183	0.8921	0.9375
Specificity	0.9884	± 0.0058	0.9812	0.9956	0.9334	± 0.0191	0.9097	0.9571
F1-score	0.9634	± 0.0143	0.9456	0.9812	0.9116	± 0.0200	0.8867	0.9365

The InceptionResNet-v2 architecture displayed strong overall performance, outperforming Inception-v3 in all key metrics, particularly for cavitated teeth, where it achieved higher precision (0.9334 vs 0.9102), recall (0.8908 vs 0.8166), and F1-score (0.9116 vs 0.8602) than Inception-v3. For non-cavitated teeth, both architectures performed similarly, but InceptionResNet-v2 was slightly more advantageous for recall (0.9370 vs 0.9154) and F1-score (0.9634 vs 0.9508). InceptionResNet-v2 had high accuracy (96.22% for non-cavitated and 91.48% for cavitated teeth), which indicates its strong classification ability. However, its performance was more stable for non-cavitated teeth—standard deviations were low, and confidence intervals were narrow, which suggests high reliability. In contrast, for cavitated teeth, InceptionResNet-v2 showed more variability, with lower recall (89.08%) and a wider confidence interval (0.8582–0.9234), which indicates that it could occasionally misclassify cavitated teeth as healthy. InceptionResNet-v2 also exhibited high specificity (93.34% for cavitated and 98.84% for non-cavitated teeth), which means that it rarely misidentified healthy teeth as cavitated, but it had more difficulty distinguishing cavitated teeth correctly. Overall, while InceptionResNet-v2 performed well, particularly for non-cavitated teeth, its robustness varied by class—it detected healthy teeth more consistently and identified cavitated teeth with more uncertainty.

### 3.3. A proof of concept

Healthcare institutions face challenges in managing vast amounts of patient data daily. As populations grow and healthcare demands rise, data fragmentation complicates retrieval and analysis, while high patient volumes strain practitioners. At FORP, researchers in dentistry seek to correlate contextual data to study epidemiology at the city-level and to allocate resources. However, lack of advanced integrated storage solutions and reliance on manual procedures make this process inefficient. Consequently, large-scale initiatives can take up to a decade to complete a public health analysis under these conditions [63]. Given that AI can potentially optimize clinical workflows, particularly in image-based diagnosis [64], we are developing a proof of concept dentistry-focused healthcare application tailored to [65]:

- assist radiologists or imaging specialists in classifying panoramic dental images to aid dental decision-making. This will be achieved by using an interactive interface capable of configuring AI models to run images to observe image classifications.
- analyze image sets and metadata to infer epidemiological knowledge efficiently, addressing the cost of epidemiological studies. Collecting images and metadata supports the application to present ways to relate oral health data of a specific studied population.
- provide collections of data and images for scientific research, including the distribution of our dataset [48] to researchers in Brazil and abroad.

**Fig 4:**
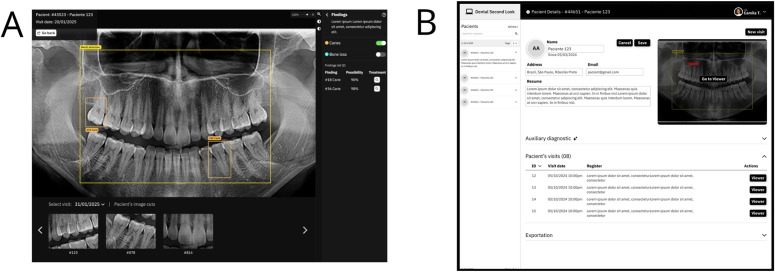
User interfaces (UI). **(A)** UI for tooth cavity detection renders **(B)** Dashboard of patient’s records.

Our proof of concept is being developed through interviews and feedback with professionals, to ensure that it evolves with research advancements and practical needs. In the near future, we aim to present a viable concept for an AI-powered application that addresses a key issue in dental health: the time-consuming process of analyzing image-based data in odontology. The goal is to showcase how these models can assist professionals by providing a suite of tools for faster analysis and deeper insights. The tool will be an automated system that analyzes PANs through a suite of CNN architectures to support clinical decision-making. Nowadays, we offer an informed viewing process of annotated PANs (see Fig 4A) and patient data management (see Fig 4B). In Fig 4A, the visualizer allows a specialist to work directly with images, using features like zoom and overlay toggles to view and edit information represented by shapes or boxes (e.g., tooth cavity detection). The viewer user interface allows the panoramic image to be visualized in detail and features menu tabs (top right side) that provide access to various configuration options and annotation features. A summary panel (middle right) compiles the most significant clinical observations for each tooth, while a comparison area (bottom) enables users to contrast the current image with previous patient records, allowing the dentist to monitor the progression of diagnoses and treatments. Each rendered image can be labeled and resized to facilitate more in-depth examination and to improve clinical documentation. In the top right corner, there is a specific tooth (using the numbering model) which indicates the quality of every tooth based on all occurrences.

In Fig 4B, the dashboard complements this by compiling all patient records into a single interface for easier management. A key feature is its advanced search capability (currently under development), designed to support complex data queries, streamline analysis, and facilitate easy data export for managers and health professionals. The core idea is to use incremental development and validation to refine the overall concept—identifying features and elements that could add value to a future robust clinical tool focused on diagnostic support and data management.

The architecture follows an MVCS model (Models, Views, Controllers, Services), with React.js views deployed via Next.js. It handles user interactions and renders them by using HTML5 Canvas and Treejs-React. A Python WSGI-Flask server-side communicates via a REST API by using FormData for images and JSON for metadata. The application currently being developed is available at [https://inredd.com.br] [64]. For the DL components, their integration relies on PyTorch’s tracing and scripting serialization capabilities (for architectures that are not developed in the PyTorch framework, we converted them by using ONNX patterns), which allow us to store a neural network’s complete checkpoint state, including weights, architectural configuration, and processing dependencies, within a single archive or multiple combined archives. This approach ensures a standard way to provide portability across the development and production environments.

**Fig 5:**
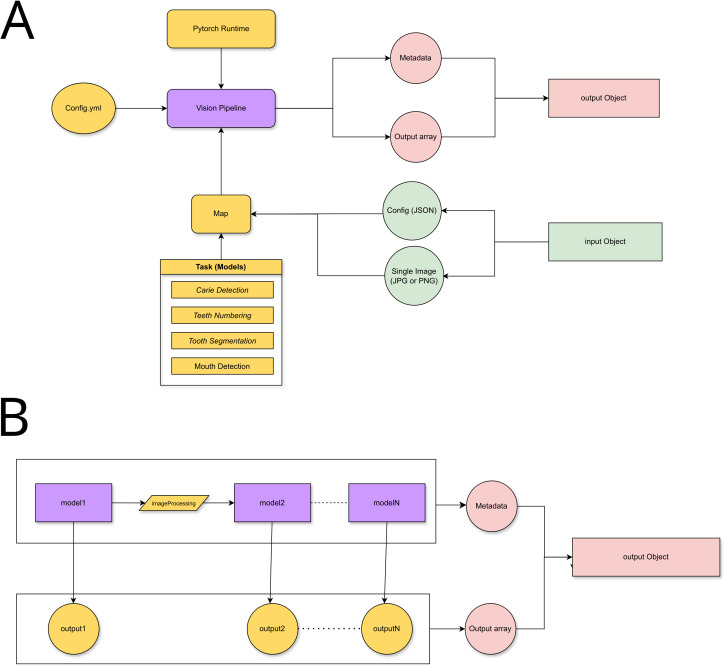
Proof of concept implementation. **(A)** Server-side (service layer) DL processing architecture **(B)** Vision Pipeline with modular DL processing.

At the service layer (see Fig 5A), each architecture is encapsulated in a class that loads the serialization archive and provides task-specific functionality (by using a mapping component), adhering to the Strategy Pattern (called “Tasks” or “Steps”). This approach enables architectures to be dynamically selected for a given processing request, on the basis of an input object defined by the controller.

A hierarchical classification process, configured in a YAML file, determines the execution order and architecture dependencies, as described in [64]. This guarantees extensibility for future implementations. With the execution context and loaded architectures, a Vision Pipeline is mounted on the basis of the chain of responsibility pattern. This Pipeline is structured as a processing flow (see Fig 5B) that produces modular results of each step and a combined output object in the JSON format, which aggregates the inference results and metadata. The user interface (Views) is designed by using components, abstracting code modules that can be rendered, and picking every architecture in the output object to map to a Drawable component, enabling it to render the DL result (see Fig 4A - center) or to use it in an informative way like numbering in the toolbox menu view. Albeit still under development, the system aims to enhance clinical support in panoramic radiograph analysis by providing an integrated environment that merges advanced machine learning methods with an interface designed to streamline workflows in dental practices.

## 4. Related work

We have conducted a systematic literature review [56] to identify prior research on using DL for dental radiographs. Our work followed current trends in DL techniques to identify tooth and caries and achieved **higher precision** than most related studies discussed in [56]. Beyond performance gains, our approach stands out because it prioritizes the development of accessible solutions and reproducible resources, as demonstrated by our proof of concept (summarized above and presented in [64]). By addressing critical gaps, our study plays a pivotal role in bridging the gap between innovative research and real-world applications, fostering advancements in dental diagnosis [64]. We focused on tasks related to teeth: detection, segmentation, numbering, and classification (caries and restorations). We present a summary of the most relevant findings.

Our method achieved higher precision and recall than [25] because we used separate architecture modules for **tooth numbering and tooth cavity detection** and employed PANs, which capture all teeth in one image. A six-layer Convolutional Neural Network, utilizing a different numbering approach, achieved a comparable precision of 0.891 [32]. A study reported slightly lower scores (0.978) on a larger dataset, suggesting that using more data has the potential for further improving our application [66]. Another study using similar images and data augmentation also achieved lower overall performance (0.885 accuracy) [36] compared to our approach. Estai et al. (2022) used panoramic radiographs to detect teeth by using the FDI system. Their approach closely resembles our modular system: (i) it identifies the oral region; (ii) locates the teeth; and (iii) numbers them according to the FDI system [67]. These authors employed a distinct neural network architecture for each step. Specifically, they used a VGG-16 architecture for the numbering task, achieving precision, recall, and F1-score of 0.98. In our study, the best results for precision, recall, and F1-score were 0.979, 0.978, and 0.978, respectively, demonstrating performance comparable to the state of the art. Mima et al. (2022) also used panoramic radiographs to number teeth. First, they applied a Faster R-CNN to identify the oral region [68]. Then, another Faster R-CNN divided this region into six parts, with each part containing a specific type of tooth. Finally, a third Faster R-CNN identified and numbered the teeth within these areas. This approach achieved an accuracy of 0.917, outperforming the application of a single Faster R-CNN on the entire oral region, which gave an accuracy of 0.888. Our study handled mouth region detection and tooth localization in separate modules (Tasks 1 and 2), which are not covered here. Task 1 uses a Faster R-CNN to detect the oral region, while task 2 employs a Mask R-CNN to locate the teeth.

For **decayed tooth identification** [23], achieved good results by using periapical radiographs and Inception-v3 (accuracy of 0.890 for premolars), but our approach using PANs and Inception-v3 or InceptionResNet-v2 achieved even better overall precision (0.910 and 0.939, respectively) for tooth cavity which can indicate dental caries. [44] classified teeth as “normal” or “anomalous” with slightly higher scores (precision of 0.987) by using a broader anomalous category. Our approach achieved good results (precision of 0.963) with a simpler architecture and larger dataset. [26] focused on predicting caries treatment by using intraoral radiographs, achieving high precision (0.990) for non-cavitated teeth. Our study aimed to simplify cavitated tooth identification (precision of 0.991 for non-cavitated teeth) by using PANs. [17] achieved lower accuracy (0.733) for decayed teeth by using intraoral radiographs and a simpler CNN architecture. We did not focus on the dental caries stage of development, specially because it would involve clinical exam, but simply on cavitated *vs*. non-cavitated.

Kunt et al. [69] trained a CNN to detect caries on bitewing images and obtained an F1-score of 0.859. Likewise, Pérez de Frutos et al. [70] used bitewings and deep learning for binary caries classification (carious vs. non-carious regions), reporting a precision of 0.88 and a recall of 0.83. Although our model used panoramic radiographs—less sensitive than bitewings for early or proximal lesions—we achieved a higher F1-score (0.937) and precision (0.939) by classifying entire teeth rather than isolated regions. This difference in task scope and image modality highlights not only the versatility of our approach but also its potential for broad, scalable screening applications in routine dental practice.

Bui, Hamamoto, and Paing (2022) proposed a system to screen dental caries efficiently and accurately [71]. Their study used the YOLO (You Only Look Once) network to detect and to locate potential carious areas in panoramic radiographs. Then, they classified regions by employing a deep learning model that was trained to confirm the presence of caries. An ensemble learning approach, combining multiple CNN models, improved accuracy and provided accuracy of 0.936, recall of 0.939, and specificity of 0.933, making the method promising for practical implementation. By applying InceptionResNet-v2, we obtained accuracy of 0.939, recall of 0.914, and specificity of 0.961. Thus, Bui et al.’s study, with higher recall, detected caries more effectively, thereby reducing false negatives, while we achieved higher specificity and identified healthy teeth better, which minimized false positives and potential misdiagnoses. Li et al. developed an automated method to detect dental caries and periapical periodontitis by using a modified ResNet-18 backbone on periapical intraoral radiographs [72]. They classified the images into crown and root regions, and caries were annotated by certified dentists. Three junior dentists initially evaluated 400 teeth and reanalyzed the same images two weeks later with the aid of the researchers’ architecture, to achieve improved diagnosis. The architecture achieved a recall of 0.835 and an F1-score of 0.829. Although intraoral radiographs facilitate caries identification, the results we obtained by using extraoral radiographs and InceptionResNet-v2 were promising: recall was 0.914, and F1-score was 0.937. The ideas proposed by [72] may help to enhance dental diagnoses and to classify caries by location.

Compared to related studies, our work has demonstrated competitive performance of our application in terms of tooth numbering and tooth cavity identification.

## 5. Limitations and future directions

Despite promising results, this study has limitations that inform both interpretation and future research.

Although class weighting was considered, it was not implemented, as strategies such as data augmentation, macro-averaged metrics, and expert-informed sampling were prioritized. Nonetheless, future work should explore cost-sensitive learning more systematically in both tooth numbering and tooth cavity detection tasks, especially to address subtle intra-class imbalances and improve performance on underrepresented categories.

In the tooth numbering task, a key limitation was the restricted dataset size and inherent imbalance among tooth classes. Data augmentation improved generalization, but a more extensive and heterogeneous dataset could further enhance reliability. All radiographs were acquired from panoramic X-ray devices at FORP, limiting representativeness. To support transparency and reproducibility, our dataset is publicly available. For tooth cavity detection, although binary class balance was achieved (“cavitated” vs. “non-cavitated”), imbalances remained across individual tooth types and lesion severities. Future datasets should ensure more uniform coverage across all 32 dental classes and incorporate lesion grading (e.g., as proposed by Moran et al., 2021) to support more personalized treatment planning.

This study did not explore preprocessing techniques such as noise reduction or image normalization, which may improve consistency and diagnostic accuracy. Additionally, future architectures might benefit from attention mechanisms to better capture anatomical variations, particularly in complex regions. Another limitation involves the exclusive use of panoramic radiographs for tooth cavity detection. While PANs are widely used and offer broad coverage, they are not the gold standard for detecting early or proximal lesions due to lower spatial resolution and anatomical overlap. In contrast, bitewing intraoral images offer higher sensitivity. Thus, results should be interpreted with caution in terms of generalizability. Nevertheless, our method shows promise as a screening or triage tool, especially in settings where only PANs are available.

Finally, although InceptionResNet-v2 consistently showed better average performance than Inception-v3 in both tasks, statistical significance was not reached after correction for multiple comparisons—likely due to the small number of folds (n = 5). Future studies should involve larger datasets, more folds, and cross-institutional validation to strengthen the observed trends and enhance clinical applicability. From a clinical perspective, improved classification in anatomically complex regions—especially posterior teeth—can support more accurate diagnostics and treatment planning. Conversely, misclassifications in these areas, particularly false negatives, could lead to missed pathology or delayed interventions. Thus, optimizing model performance in such regions remains both a technical and clinical priority.

## 6. Final remarks

Our research proposes a high-performance system for numbering teeth (FDI system) and identifying cavitated teeth in panoramic radiographs (PANs) by using deep convolutional neural networks (CNNs) within a modular, multi-task framework designed specifically for analyzing PANs.

InceptionResNet-v2 consistently achieved higher average performance than Inception-v3 across all key metrics in both tasks, with faster convergence (10–25 vs. 35–50 epochs). Although differences were not statistically significant after correction for multiple comparisons, the directional trends favor InceptionResNet-v2, particularly for anatomically complex regions such as posterior teeth. Performance was enhanced by incorporating anatomical context through bounding box expansion, rigorous expert annotation, data augmentation, and a diverse set of hyperparameters.

For the cavitated tooth identification, InceptionResNet-v2 also outperformed Inception-v3, demonstrating better class recognition between “cavitated” and “non-cavitated” teeth. High precision values indicate the architectures excel at identifying true positives while minimizing false positives. High specificity values show strong performance in classifying negative cases. Limitations include imbalanced tooth types and varying degrees of cavitation within the dataset. Future work should focus on more uniform distributions and consider cavity severity to improve treatment planning. Despite these limitations, combining datasets with diverse annotations yielded promising results.

The modular computational system, developed jointly with Carneiro et al. [47], integrates tooth numbering and tooth cavitated detection for various dental applications like treatment planning and caries monitoring. This modular framework allows for future expansions, including tasks like restoration detection or impacted tooth identification. By using different CNNs for specific analyses, the system adapts to various needs and holds promise for automating large-scale data analysis in dentistry, improving report generation, and populating dental records. Moreover, this system can act as a valuable second opinion for dentists, reducing errors and aiding learning through AI comparison.

Overall, this work demonstrates the potential of deep learning to assist in large-scale dental radiographic analysis. Future research should address dataset limitations and pursue expanded functionalities to further enhance clinical relevance.
